# Evaluation of stress distribution in bone and three-unit fixed implant-supported prostheses with zirconia and titanium abutments: A 3D finite element analysis

**DOI:** 10.15171/joddd.2018.040

**Published:** 2018-12-19

**Authors:** Fahimeh Hamedirad, Tahereh Ghaffari, Navideh Mehanfar

**Affiliations:** ^1^Department of Prosthodontics, Babol University of Medical Sciences, Babol, Iran; ^2^Department of Prosthodontics, Faculty of Dentistry, Tabriz University of Medical Sciences, Tabriz, Iran

**Keywords:** Dental implant, implant-supported dental prosthesis, finite element analysis, stress distribution

## Abstract

***Background.*** For esthetic considerations in anterior regions, abutments with high-strength ceramics such as alumina and zirconia have been developed as substitutes for titanium abutments. The present study was designed to investigate the distribution of stress in prosthesis and bone components of an implant-supported FPD with different abutments by using 3D finite element analysis.

***Methods.*** Ceramic FPDs were made from the canine to the upper left second premolar with titanium fixtures. In order to investigate the stress distribution, forces of 100 and 300 N were applied at angles of 0, 15 and 35 degrees to the central fossa of the second premolar and pontic, as well as the cingulum of the canine crown. Force loading was static. After analyzing the mechanical properties of the materials, boundary conditions and loading were performed according to the existing averages, and subsequently, the results obtained from this analysis were analyzed.

***Results.*** The highest level of stress was observed in the distal crest of the posterior implant (23.20 MPa) under lateral forces (15 and 35 degrees) in a model with both titanium abutments.

***Conclusion.*** Lateral forces induced higher accumulation of stress in the implant and surrounding bone, while abutment change did not affect the distribution of stress.

## Introduction

Dental implants are prescribed for reconstruction of completely or partially edentulous patients. Replacement of the tooth in the anterior regions requires esthetic considerations. Despite numerous modifications in the construction and design of metal abutments, the challenges with the metal components of these abutments persist.^1^ To surmount this challenge, abutments with high-strength ceramics such as alumina and zirconia have been developed as substitutes for titanium abutments. Ceramic abutments have advantages such as esthetics, less discoloration in the mucosa around the implant and less bacterial accumulation compared to titanium abutments. However, poor mechanical properties of this material such as brittleness and low resistance against tensile forces have limited its application. Zirconia has been accepted as a suitable ceramic for construction of abutments with the highest fracture resistance among various types of ceramics.^2^

The results of a study in 2010 revealed that zirconia abutments reinforced with titanium have the same efficacy as titanium abutments; hence, they can be employed as an alternative to single-unit implants in the anterior region.^3^ In several studies, the success rate of zirconia abutments has been reported to be 100% in single-unit crowns of anterior and premolar regions.^4,5^ No report has been presented in terms of the failure of zirconia abutment so far. Abutment screw loosening is one of the few technical problems associated with the use of zirconia abutments, and this problem is similar to that of titanium abutments.^2^ In one clinical study, zirconia abutments had proper stability for single-unit implant-supported restorations; the level of bone margin around the implant was stable and the soft tissue was completely healthy. In this study, zirconia abutment induced less compression and von Mises stress on implant and cortical bone compared to titanium abutment.^1^

Another study evaluated the effect of the use of different implant abutments supporting single-unit restorations on the distribution of stress in bone. The results showed that different abutments had no effect on the distribution of force in bone. However, lower stresses on the retentive screw were only observed in zirconia abutments.^6^

The results of a study in 2013 showed that the distribution of stresses in titanium and zirconia implants was very similar; the only difference was that lateral forces created less stress on the titanium implant and prosthetic cores.^7^

In several studies on implant-supported prosthesis, one type of abutment was investigated; if one of the abutments is placed in the esthetic region, it can be made from zirconia abutments as compared to posterior titanium abutment for better strength; however, no study has been performed on the advantages and disadvantages of the use of zirconia and titanium combined abutments. Consequently, the present study was designed to investigate the distribution of stress in the prosthetic and bone components of an implant-supported FPD with different abutments.

## Methods

Ceramic FPDs were made on abutments from the canine to the upper left second premolar with titanium fixtures. In the first sample, both abutments were made from titanium; in the second sample, the anterior abutment was made from zirconia and the posterior abutment was made from titanium and in the third sample, both abutments were made from zirconia. Finally, a three-unit FPD with a zirconia frame and feldspathic porcelain veneer was designed on all of them.

The upper jaw was simulated using CT images (conventional tomography) of an edentulous person in the canine region and the first and second upper left premolars with Cl I occlusion of angle classification and without any craniofacial deformity. Bone modeling was carried out in the upper left canine and upper second left premolar using the CBCT (Mimics: Materialise Interactive Medical Image Control System; Leuven Belgium). In this study, the quality of the bone was type D3 and the thickness of the cortical bone on the buccal and lingual aspects was considered as 1 mm on the trabecular bone nucleus.^8^

For modeling, a titanium cylindrical implant (Astra tech AB, Stockholm, Sweden) with a length of 13 mm and a diameter of 4.5 mm was employed. Abutment and abutment screw models, with a length of 8 mm and a diameter of 4.5 mm and a gingival height of 1.5 mm, were used. Implants, abutments, abutment screws and bridges were scanned using a 3D scanner (GOM Measurement device, Braunschweig, Germany (ATOSII)). The measured data were transferred to the modeling software (3D CAD 2010 Solid Work Conccord US) to provide solid models.^9^

In order to make an all-ceramic FPD, a zirconia core was prepared by CAD-CAM with an 0.8-mm thickness in the axial walls and 1 mm in the occlusal region and veneered using feldspathic porcelain. The cross-sectional size of the connectors was 3.5×3.5 mm, and the pontic in the first premolar tooth was designed as a ridge lap model.^10^

The interface of the implant to the bone was modeled as a complete osseointegration.^11^

All the environmental nodes of the model were considered to be constant and had no freedom of movement in order to prevent the movement of the model during force application.

Table 1 presents the mechanical properties of different parts of the model.^1,10^

**Table 1:** The mechanical properties of different parts of the model

**Materials**	**Young’s modulus (GPa)**	**Poisson ratio**
**Zirconia abutment and core**	200	0.31
**Trabecular bone**	1.37	0.3
**Cortical bone**	13.7	0.3
**Titanium abutment and implant and screw**	115	0.35
**Glass ionomer cement**	7.56	0.35
**Feldspathic porcelain**	68.9	0.28

In order to investigate stress distribution, forces of 100 and 300 N were applied at angles of 0, 15 and 35 degrees to the central fossa of the second premolar and pontic, as well as the cingulum of the canine crown. Force loading was static.^11^

The scanned data were transferred to the modeling software (Auto CAD2010, Solid Works 2012, Rapid Form 2006) to provide solid models. The fixture was placed inside the bone and the abutment and the abutment screw and bridge were placed on it. The prepared mounted model for mathematical analysis was transferred to ABAQUS software (version 6.13) (Hibbitt, Karlsson, and Sorensen, Inc. Rhode, Iceland). The number of elements forming the model was 834494 and the tetrahedral type and number of nodes was 178947. For the cement layer, a thin layer with 20 µm of thickness was meshed and the characteristics of the glass-ionomer cement were registered in the software.^12^ After analyzing the mechanical properties of the materials, boundary conditions and loading was performed according to the existing averages, and subsequently, the results were analyzed.

## Results

**Figure 1:**
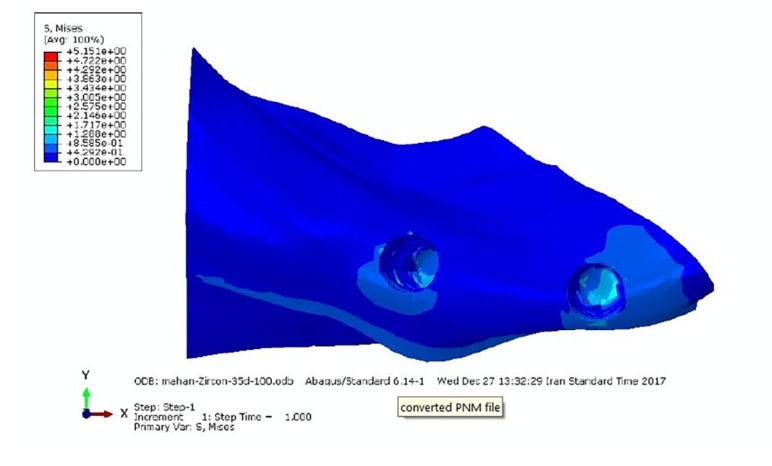
Distribution of von Misses stress in cancellous bone by applying 100-N force at 35- degree angle.

**Figure 2:**
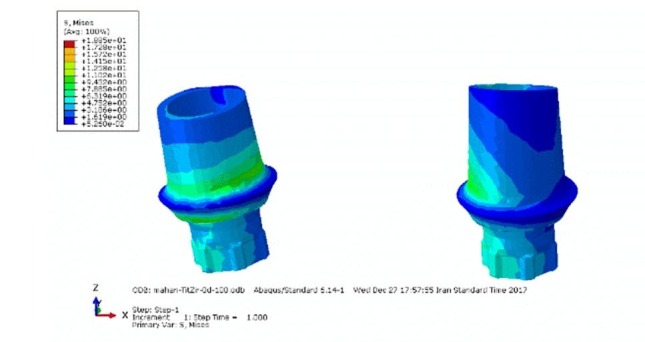
Distribution of von Misses stress in zirconia anterior abutment and titanium posterior abutment by applying 100-N force at zero-degree angle.

With due attention to Tables 2 and 3 and Figures 1 and 2, the following results were obtained:

**Table 2:** Stress levels in one third part of cervical trabecular bone(a), apex of implants in trabecular bone(b) and crest of cortical bone using 100 N force

**Angle**	**Abutments**	**Posterior**			**Anterior**		
		**a**	**b**	**c**	**a**	**b**	**c**
**0-degree angle**	**Ti-Ti**	0.51	0.51	10.59	0.51	0.51	1.92
	**Ti-Zr**	0.64	0.51	10.6	0.64	0.51	1.92
	**Zr-zr**	1.3	0.51	10.46	1.3	0.51	1.9
**15-degree angle**	**Ti-Ti**	0.51	0.64	20.23	0.51	0.64	7.3
	**Ti-Zr**	0.64	0.63	19.26	0.64	0.63	7
	**Zr-Zr**	0.85	0.64	19.67	0.85	0.64	7.1
**35-degree angle**	**Ti-Ti**	0.51	1.3	20.23	0.51	1.3	7.3
	**Ti-Zr**	0.63	0.84	19.26	0.63	0.84	7
	**Zr-Zr**	0.84	0.85	19.67	0.84	0.85	7.1

**Table 3:** Stress levels in one third part of cervical trabecular bone(a), apex of implants in trabecular bone(b) and crest of cortical bone using 100 N force

**Angle**	**Abutments**	**Posterior**			**Anterior**		
		**a**	**b**	**c**	**a**	**b**	**c**
**0-degree angle**	Ti-Ti	1.54	2.23	34.7	1.54	2.32	8.6
	Zr-Zr	2.31	2.31	34.2	2.31	2.31	8.5
	Ti-Zr	1.54	2.3	31.84	1.54	2.3	8.6
**15-degree angle**	Ti-Ti	1.92	2.89	47.27	1.92	2.89	11.82
	Zr-Zr	1.92	2.8	47.27	1.92	2.8	11.82
	Ti-Zr	1.91	2.86	42.54	1.91	2.86	15.47
**35-degree angle**	Ti-Ti	2.6	3.9	60.74	2.6	3.9	22.09
	Zr-Zr	2.57	3.86	48.33	2.57	3.86	21.48
	Ti-Zr	3.8	3.8	63.9	2.53	3.8	26.29

In the present study, in all forms of loading, the stress transmitted from the model with both titanium abutments was a little higher compared to other models (Tables 2 and 3).

In this study, the highest level of von Mises stress as seen in Tables 2 and 3 was observed in the distal cortical crest of the posterior implant (23.20 MPa) under lateral forces (15 and 35 degrees) in a model with both titanium abutments.

The stress level increased when the angle of the lateral forces increased by 35 degrees.

The stress distribution, when a force of 300 N was applied, was similar to the force of 100 N; only the amount of von Mises stresses increased proportionate to the increase in force (Table 3 and Figure 1).

Under lateral forces, the highest tensions were observed in the posterior abutment, especially in both titanium abutments (Table 4 and Figure 2).

**Table 4:** Stress levels in abutments using 100 N force (a) and 300N force (b)

**Angle**	**Abutments**	**posterior**		**Anterior**		
		**a**	**b**	**a**	**b**
**0-degree angle**	Ti-Ti	9.49	28.51	9.49	28.51
	Zr-Zr	9.45	28.68	9.45	28.68
	Ti-Zr	9.45	28.38	9.54	28.38
**15-degree angle**	Ti-Ti	23.21	69.69	19.37	58.16
	Zr-Zr	22.25	68.31	18.57	57.01
	Ti-Zr	22.75	66.82	18.99	55.77
**35-degree angle**	Ti-Ti	45.87	137.7	39.37	98.71
	Zr-Zr	43.47	134.2	31.1	96.11
	Ti-Zr	44.68	130.5	32	93.54

## Discussion

Three-dimensional finite element analysis is used to estimate stress distribution in structures subjected to mechanical loading. This technique overcomes most of the problems of primary testing methods. The dimensions and mechanical properties of the materials are easily simulated and any stress variations can be computed.^13^ Many factors such as the direction and amount of force have been shown to influence the distribution of stress.

In this study, the challenge was whether the application of force at more than one point would induce more stress in the framework and occlusal surface of the implant-supported FDP and lower stress distribution in the bone.

In the present study, in all forms of loading, the stresses transmitted from the model with both titanium abutments was higher compared to other models, though the difference in stress transmission from all the models into the bone was not notable. This difference might be due to the higher modulus of elasticity (MOE) of the zirconia abutment. MOE is one of the significant factors determining the behavior of materials. The MOE of zirconia abutment (200 GPa) is more than that of titanium abutment (110 GPa). The different elastic modulus of the implant material affects the implant−bone interface. Materials with a very low elasticity should be avoided. Implant materials must have at least an elastic modulus of 110 GPa because materials with low MOE will induce more stress in the bone.^1^

In this study, the highest level of stress was observed in the distal cortical crest of the posterior implant (23.20 MPa) under lateral forces (15 and 35 degrees) in the model with both titanium abutments. The stresses created in implants crest were based on the rigid bonds between the implants and the bones and were similar to the results of previous studies. In implant-supported fixed prostheses, the stresses from functional forces are directly transmitted to the bone, which is due to the lack of periodontal ligament, and the highest stress is found around the implant near the crest. Due to the geometric shape of the upper jaw, the anterior and posterior fixtures were not in the same direction and had an angle of 9 degree with each other. The forces applied to the bridge were parallel to the anterior implant. Therefore, the highest tensions were observed in the distal crest of the posterior abutment. However, the main factor affecting this situation was the force points on the bridge. The observed points with the highest tension around the implant were precisely consistent with the force applied on the bridge. If CAD/CAM software is employed to design the implant surgery and prosthesis, so that the location of force application is exactly along the longitudinal axis of the implants, it will yield different results as observed from the findings of the study. The values recorded under the study conditions (design, loading conditions and elastic properties of the material) was lower than the maximum compressive and tensile strengths of the cortical bone (121 MPa and 167 MPa).^1^

Kohal et al^14^ examined the distribution of stress separately in titanium and zirconia implants and the surrounding bone. The metal-ceramic crown for the titanium implant and all-ceramic crowns for zirconia implant were modeled. They reported that stress distribution in zirconia implants was very similar to that in titanium implants.The results of this study were consistent with those reported by Kohal et al,^14^ while the amount of the stresses observed around the titanium abutment with lateral and horizontal forces were higher than the zirconia abutment.

The stress level increased when the angle of the lateral forces increased by 35 degrees. This is possibly due to the bending of the components that influenced the maximum stress on the implant.^11^

Table 3. Stress levels in one third part of cervical trabecular bone(a), apex of implants in trabecular bone(b) and crest of cortical bone using 100 N force

The results of this study showed that highest von Mises stresses were found in the cortical bone. Elasticity coefficient of cortical bone was higher than trabecular bone.^6,13^ For this reason, the cortical bone was tighter and more resistant against the changes. Consequently, the amount of stress reported in the cortical bone was higher than the trabecular bone. Overall, the results of this study showed that in different types of abutments, distribution of stress to the cortical bone was almost similar. Therefore, abutments might only play a role in transferring forces to the bone, and different abutment types do not affect it.

One of the factors influencing the results of this study is how the forces were applied. If the force similar to the physiological conditions was applied to the whole surface of the bridge rather than at a point, more varied results would be obtained. In this study, the distribution of stress at angles of zero, 15 and 35 degrees for each force of 100 N and 300 N was the same in all the three models. Only the stress value increased relatively when compared to the increase in force. The causal factors, the similarity of the constraints and the geometry in both cases were similar in terms of response of the structures.

According to previous studies^15,16^ and the results of the present study, the change in the type of abutment does not induce a significant reduction in the stress in implant and the surrounding bone. This can be attributed to the low level of stress applied in the present study, and if the magnitude of the stresses on the implant is greater, the difference in stiffness and stress between the titanium and zirconia abutments will greatly increase. The results of the study conducted by Kaleli et al^17^ in 2018 showed that several layers or structures, including crown, cement layer, internal screw and abutment, play a role in the transfer of chewing forces to implants and bone. The total energy transferred to the bone−implant interface first passes through the implant abutment interface. Some of the transmitted energy is absorbed by interstitial structures. This paper explains the reasons for similar biomechanical responses in implants with abutments made of different materials.

Some inherent limitations of this study include changes in the biomechanical behavior of the components as well as changes in the type of fixture, the differences in hex abrasion of the titanium and zirconia abutments as well as time of passage. Further studies are recommended on the biomechanical behavior of the components with anterior abutments and implants from zirconia and posterior abutments and implants from titanium. In addition, evaluation of the force distribution when the fixture is made of zirconia is also suggested in order to complement the results obtained in this study.

## Conclusion

Under the limitations of this study, the following results were obtained:

- Lateral forces induced more stress accumulation.
- Material change did not affect the distribution of stress in the implant and surrounding bone.
- In all the three models, the highest stress levels were observed in all the three loading models in the distal crest of the posterior implant.
- The use of zirconia abutment slightly reduced the amount of stress transferred to the bone.

## Acknowledgments

The authors acknowledge the support of Tabriz University of Medical Sciences.

## Authors’ Contribution

All the authors were responsible for the concept and definition of intellectual content. FH and NM designed the study and performed the literature review. TG performed the experiments and drafted the manuscript. Data acquisition and analysis were performed by FH and NM. All the authors critically revised the manuscript. All the authors have read and approved the final manuscript.

## Funding

This study was supported by Tabriz University of Medical Sciences.

## Competing interests

The authors declare no competing interests with regards to the authorship and/or publication of this article.

## Ethics approval

Not applicable.
